# Negative impact of chronic obstructive pulmonary disease on the health-related quality of life of patients. Results of the EPIDEPOC study

**DOI:** 10.1186/1477-7525-4-31

**Published:** 2006-05-23

**Authors:** Pilar Carrasco Garrido, Javier de Miguel Díez, Javier Rejas Gutiérrez, Antonio Martín Centeno, Elena Gobartt Vázquez, Ángel Gil de Miguel, Marta García Carballo, Rodrigo Jiménez García

**Affiliations:** 1 Preventive Medicine and Public Health Department, Universidad Rey Juan Carlos, Alcorcón, Madrid, Spain; 2 Department of Pneumology, University Hospital Gregorio Marañón, Madrid, Spain; 3 Department of Health Outcomes Research, Medical Unit, Pfizer España, Parque Empresarial La Moraleja, Avda de Europa, 20-B, 28108 Alcobendas, Madrid, Spain; 4 Respiratory Area, Medical Unit, Pfizer España, Alcobendas, Madrid, Spain; 5 Medical Department, Boehringer Ingelheim SA, Sant Cugat del Vallés, Barcelona, Spain

## Abstract

**Background:**

COPD is currently the fourth cause of morbidity and mortality in the developed world. Patients with COPD experience a progressive deterioration and disability, which lead to a worsening in their health-related quality of life (HRQoL). The aim of this work is to assess the Health-Related Quality of Life (HRQoL) of patients with stable COPD followed in primary care and to identify possible predictors of disease.

**Methods:**

It is a multicenter, epidemiological, observational, descriptive study. Subjects of both sexes, older than 40 years and diagnosed of COPD at least 12 months before starting the study were included. Sociodemographic data, severity of disease, comorbidity, and use of health resources in the previous 12 months were collected. All patients were administered a generic quality-of-life questionnaire, the SF-12, that enables to calculate two scores, the physical (PCS-12) and the mental (MCS-12) component summary scores.

**Results:**

10,711 patients were evaluated (75.6% men, 24.4% women), with a mean age of 67.1 years (SD 9.66). The mean value of FEV_1 _was 35.9 ± 10.0%. Mean PCS-12 and MCS-12 scores were 36.0 ± 9.9 and 48.3 ± 10.9, respectively. Compared to the reference population, patients with COPD had a reduction of PCS-12, even in mild stages of the disease. The correlation with FEV_1 _was higher for PCS-12 (r = 0.38) than for MCS-12 (r = 0.12). Predictors for both HRQoL components were sex, FEV_1_, use of oxygen therapy, and number of visits to emergency rooms and hospital admissions. Other independent predictors of PCS-12 were age, body mass index and educational level.

**Conclusion:**

Patients with stable COPD show a reduction of their HRQoL, even in mild stages of the disease. The factors determining the HRQoL include sex, FEV_1_, use of oxygen therapy, and number of visits to emergency rooms and hospital admissions.

## Background

Chronic obstructive pulmonary disease (COPD) is characterized by the presence of a limitation to airflow that is not completely reversible and is associated with an abnormal inflammatory response to gases or inhaled toxic particles, mainly tobacco [1,2]. COPD is currently the fourth cause of morbidity and mortality in the developed world [1]. The IBERPOC study, carried out in 7 different geographical areas with nearly 5000 patients, has shown that in Spain the prevalence of COPD in the general population aged between 40 and 69 years is 9.1%, and is a first-order public health problem [3]. This condition originates approximately 10–12% of primary care consultations and 35–40% of those of pneumology, and causes 35% of definitive occupational disabilities and 7% of hospital admissions [4].

As the condition progresses, patients with COPD experience a progressive deterioration and disability, which lead to a worsening in their health-related quality of life (HRQoL). However, it has been confirmed that the evaluation alone of the severity of COPD, measured by the degree of reduction of the forced expiratory volume in one second (FEV_1_), does not provide sufficient information to establish the health condition perceived by the patients. The interest for HRQoL measurement in patients with COPD has grown in recent years. The fact that HRQoL is the result of the interaction of multiple physical, psychological and social factors, unique for each individual, can justify this interest from the scientific community [5]. Each of these domains can be measured with the objective of establishing the performance or health condition and the subjective perception of health. The HRQoL can be quantified through various health evaluation questionnaires, both general and specific, widely validated [6]. The former covers a broad range of dimensions, enable the comparison between groups of patients with different diseases, and facilitate the detection of problems or unexpected effects [7]. One of them is the Short Form 36 (SF-36) questionnaire [8], that has an abbreviated version (SF-12) [9,10]. Since one of the main objectives of the treatment of COPD is to improve the general health condition [1], it is important to identify possible factors determining the HRQoL of these patients. Some of these have been identified previously [11]. Relationships have been shown between HRQoL during acute exacerbation of chronic bronchitis with post-exacerbation functional status, hospital readmission for acute exacerbation or COPD and mortality [12-16].

The objective of this study was to assess the HRQoL of a group of patients with stable COPD followed in primary care and to identify the associated determinant factors.

## Methods

### Design and population of the study

This study is part of the EPIDEPOC study, a multicenter, epidemiological, observational, descriptive project, carried out in the primary care setting, with the aim of estimating the use of health resources and assessing the HRQoL of patients with stable COPD.

The recruitment of patients and the calculation of sample size correspond to that performed in the EPIDEPOC study. For the calculation of sample size, a cluster design was used, considering 3 types of variables: health centers, physicians, and medical records. Finally, it was estimated that it was necessary to include 2422 physicians, each of whom should recruit 5 patients. The screening of patients was performed at random by primary care physicians from all Spanish Autonomous Communities, whose distribution was weighed based on the population distribution of the different Autonomous Communities over the total national. The patients were recruited during a period of three months (from 1^st ^January to 31^st ^March 2003).

Subjects of both sexes, aged 40 years or older and diagnosed of COPD at least 12 months before the start of the study, were included. The diagnosis of the disease was performed according to the criteria of the Spanish Society of Pneumology and Chest Surgery (SEPAR) based on the demonstration, through a forced spirometry, of a forced expiratory volume in 1 second (FEV_1_) below 80% of the reference value and a FEV_1_/forced vital capacity (FVC) ratio below 0.7 after the bronchodilation test. The severity of the disease was rated at three levels according to the FEV_1 _value: mild (FEV_1 _60–80% of the reference value), moderate (FEV_1 _40–59% of the reference value) and severe (FEV_1 _below 40% of the reference value), in accordance with the SEPAR criteria [17].

Individuals suffering at the time of the study a neurological or psychiatric disease precluding measurement were excluded. Patients with an acute worsening of their COPD in the previous month were also excluded. An acute worsening was considered to be the occurrence of an impairment of the clinical condition of the patient characterized by an increased baseline dyspnea, purulent sputum, increased volume of sputum or any combination of these symptoms, of acute onset, and requiring a change in the regular medication of the patient [18].

The study was approved by the Ethics Committee of the Foundation Hospital Alcorcón, and all patients gave their oral consent to participate in it. All the information obtained will be considered as confidential and disclosed and kept following the regular safety standards (Organic Act 15/1999, of 13 December on the Protection of personal data).

### Assessment of the patients

A single visit was completed, collecting in all cases the sociodemographic data, severity of the disease, comorbidity, and utilization of health resources in the previous 12 months. All patients were administered the SF-12 quality of life questionnaire, an abbreviated version of the SF-36 health questionnaire that contains 12 items [9]. These 12 items explain more than 90% of the variance of the physical and mental component scores of the SF-36. From them two scores can be calculated, the physical (PCS-12) and the mental (PCS-12) component summary, using a value of 50 with a standard deviation of 10 as reference population. In this study, the general Spanish adult population has been used as reference [19]. The SF-12 is scored from 0 to 100 so that, the higher the score, the better the health condition.

### Statistical analysis

The analysis of the data was carried out through the statistical package SPSS 11.0 for Windows. The qualitative variables were described as frequency and percentage and the quantitative variables as mean, standard deviation, minimum and maximum. To analyze the relationship between the qualitative variables the χ^2 ^Pearson test was used. ANCOVA and ANOVA models with sex, age and disease severity as covariates were applied for between group comparisons. Bonferroni adjustment was applied for multiple comparisons. To evaluate the correlation among the values of the quantitative data, the Pearson's correlation coefficient was used. Finally, a multiple regression analysis was performed using as dependent variable the physical or mental component of the HRQoL and as independent variables: age, sex, smoking, size of the habitat, educational level, body mass index, FEV_1_, treatment with oxygen therapy, visits to emergency rooms and hospital admissions. Independent variables included in the model were chosen from those showing significance differences in the bivariate analysis or deemed to be potential confounders. A value of p < 0.05 was considered to be significant.

## Results

The number of physicians participating in the study was 2,377, which enabled to recruit a total of 10,711 patients (75.6% men, 24.4% women), with a mean age of 67.1 years (SD 9.66), and a mean body mass index of 27.6 (4.0) kg/m^2^. Table 1 shows the sociodemographic and clinical characteristics of the sample studied. The mean FEV_1 _value was 35.9 ± 10.0%. The severity of the disease was mild in 35.5% of the cases, moderate in 53.4%, and severe in 11.2%. The concomitant conditions most frequently detected were hypertension (47.7%), hypercholesterolemia (41.3%), anxiety (22.2%), heart disease (18.8%), gastroduodenal ulcer (17.4%), diabetes (16.9%), and depression (12.8%). With regard to the use of health resources in the previous year, the mean values were: visits to primary care physician 6.66 (SD 5.71), visits to pneumologist 1.43 (SD 1.52), visits to emergency rooms 1.60 (SD 2.71), and hospital admissions 0.50 (SD 1.17).

**Table 1:** Sociodemographic and clinical characteristics of patients

**Characteristic (no. of patients)**	
**Total no. of patients**	**10,711**
Age (years) * (8665)	**64.1 ± 9.7 (40; 98)**
**Age groups (8862)**	
40–54 years	**963 (10.9)**
55–64 years	**2,249 (25.4)**
65–74 years	**3,669 (41.4)**
>75 years	**1,981 (22.4)**
**Sex (man) (10620)**	**8,030 (75.6)**
**Residence population (10341)**	
<10,000 inhabitants	**3,022 (29.2)**
10,000–100,000	**3,449 (33.4)**
>100,000	**3,870 (37.4)**
**Educational level (9018)**	
No studies	**1,852 (20.5)**
Primary	**5,111 (56.7)**
Secondary	**1,582 (17.5)**
University	**473 (5.2)**
**Smoking (10649)**	
Never	**2,468 (23.2)**
Ex-smoker	**6,153 (57.8)**
Active smoker	**2,028 (19.0)**
**FEV_1 _(9963)**	**35.9 ± 10.0 (4;80)**
**COPD Severity (9963)**	
Mild	**3,634 (35.5)**
Moderate	**5,471 (53,4)**
Severe	**1,146 (11.2)**
Visits to E.R. * (9505)	**1.4 ± 1.9 (0; 12)**
No. of hospital admissions * (8670)	**0.5 ± 0.9 (0; 11)**
**Oxygen therapy (10007)**	**1351 (13.5)**
**Associated comorbidity**	
Hypertension (9876)	**4,706 (47.7)**
Hypercholesterolemia	**3,995 (41.3)**
Diabetes (9453)	**1,598 (16.9)**
Heart disease (9390)	**1,770 (18,8)**
Ulcer (9425)	**1,637 (17.4)**
Depression (9333)	**1,196 (12,8)**
Anxiety (9397)	**2,084 (19.5)**

Value expressed as frequencies (percentages)

* Value expressed as mean ± standard deviation (minimum; maximum).

**Figure 1:**
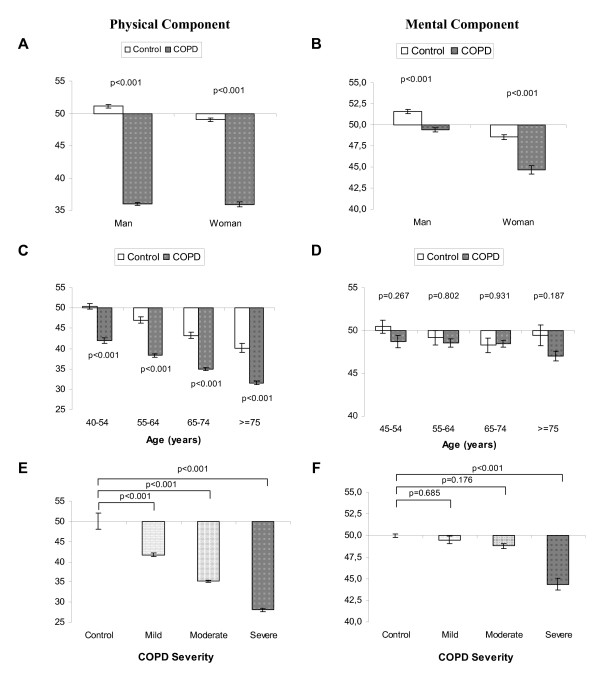
Relationship of quality of life to sex, age, and severity of COPD.

**Figure 2:**
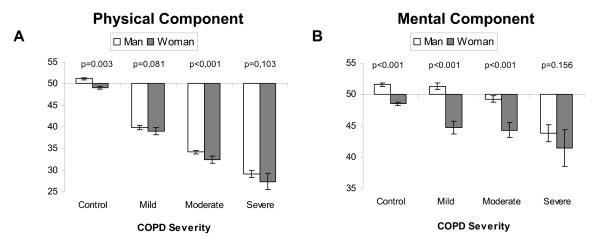
Relationship between quality of life, sex, and degree of airflow obstruction.

The mean PCS-12 and MCS-12 scores were 36.0 ± 9.9 and 48.3 ± 10.9, respectively. As compared to the reference population, patients with COPD had a reduction of PCS-12, regardless of sex, age range or degree of airflow obstruction. However, a significant decrease was only seen in MCS-12, vs the reference population, in women and patients with severe COPD, with no differences in this parameter related to age (Figure 1). Figure 2 shows the relationship between quality of life, sex, and degree of airflow obstruction. With regard to the PCS-12, significant differences were only detected between men and women in patients with moderate COPD. With regard to MCS-12, differences were found between the two sexes in patients with mild and moderate COPD, but not in patients with severe COPD.

The use of oxygen therapy was associated with a significant reduction of the quality of life, in both PCS-12 and MCS-12. Table 2 shows the distribution of the mean scores of both parameters based on the use of oxygen therapy and the variables related to utilization of health resources.

**Table 2:** Physical (PCS-12) and mental (MCS-12) component summaries related to the use of oxygen therapy and the utilization of health resources in the past year

	**No. of cases**	**PCS-12**	**MCS-12**
**Oxygen therapy:**			
No	3,131 (84.8%)	32.4 (31.3; 33.5)	45.8 (44.4; 47.2)
Yes	562 (15.2%)	30.3 (29.1; 31.6)	43.7 (42.1; 45.3)
P		0.019	0.049
**No of visits to primary care:**			
1 to 5	1,941 (52.3%)	32.9 (31.3; 34.5)	46.4 (44.3; 48.4)
5 to 10	1,140 (30.9%)	31.0 (29.5; 32.4)	44.2 (42.3;46.0)
Over 10	612 (16.6%)	31.0 (29.7; 32.3)	44.6 (42.9; 46.2)
P		0.130	0.241
**No. of visits to pneumology:**			
None	883 (23.9%)	32.8 (30.9; 34.8)	48.7 (46.2; 51.1)
1 to 4	2,711 (73.4%)	31.1 (30.3; 32.0)	44.7 (43.6; 45.8)
Over 4	99 (2.7%)	31.0 (28.9; 33.1)	41.6 (38.9; 44.2)
P		0.277	0,000
**No. of visits to E.R.:**			
None	1,481 (40.1%)	34.8 (32.7; 36.9)	48.2 (45.5; 50.8)
1 to 2	1,508 (40.8%)	31.5 (30.0; 33.1)	45.6 (43.6; 47.6)
3 to 4	466 (12.6%)	30.2 (28.8; 31.7)	45.0 (43.2; 46.8)
Over 4	238 (6.4%)	30.9 (29.2; 32.7)	41.8 (39.6; 44.0)
P		0.004	0.003
**No. of hospital admissions:**			
None	2,544 (68.9%)	33.7 (32.5;35.0)	47.4 (45.8; 49.0)
1	755 (20.4%)	32.1 (30.5; 33.7)	43.1 (41.1; 45.1)
2	257 (7.0%)	29.2 (27.4; 31.1)	44.3 (42.1; 46.6)
Over 2	137 (3.7%)	29.5 (27.2; 31.7)	43.8 (41.0; 46.6)
P		0.000	0.006

Variables expressed as mean (95% CI).

PCS-12 was correlated positively to FEV_1 _(r = 0.38, p < 0,001), however the correlation with the mental component (MCS-12) was lower (r = 0.12). Tables 3 and 4 show the factors determining physical and mental components of the HRQoL, respectively. The determinant factors of MCS-12 were sex, FEV_1_, use of oxygen therapy, and number of visits to emergency rooms and hospital admissions. The PCS-12 was also determined by age, Body Mass Index and educational level. Smoking was not significantly related to any of the components of HRQoL.

**Table 3:** Coefficients of the multiple linear regression equation for the final physical component of quality of life

**Independent variables**	**Coefficients (95% CI)**		**Standard coefficients**	**Sig.**
**Constant**	48.6	(44,8; 52,3)		<0.001
**Age**	-0.25	(-0.28; -0.27)	-0.233	<0.001
**Sex**	-1.21	(-1.91; -0.51)	-0.050	0.001
**Educational level**	1.22	(0,82; 1,62)	0.092	<0.001
**FEV_1_**	0.019	(0,16; 0,26)	0.226	<0.001
**Oxygen therapy**	-2.84	(-3.75; -1.93)	-0.101	<0.001
**Body Mass Index**	-0.17	(-0.24; -0.10)	-0.068	<0.001
≤ **2 visits to E.R.**	-0.84	(-1.02; -0.66)	-0.162	<0.001
≤ **2 hospital admissions**	-0.93	(-1.31; -0.54)	-0.086	<0.001

R^2 ^= 0.31

Dependent variable: final physical component.

Variables excluded: size of the habitat, smoking

**Table 4:** Coefficients of the multiple linear regression equation for the final mental component of quality of life

**Independent variables**	Coefficients (95% CI)		Standard coefficients	Sig.
**Constant**	52.6	(50.5; 54.8)		<0.001
**Sex**	-5.71	(-6.57; -4.84)	-0.21	<0.001
**FEV_1_**	0.08	(0.04; 0.11)	0.09	<0.001
**Oxygen therapy**	-2.25	(-3.38; -1.13)	-0.07	<0.001
**≤ 2 visits to E.R.**	-0.63	(-0.86; -0.40)	-0.11	<0.001
**≤ 2 hospital admissions**	-1.01	(-1.49; -0.53)	-0.09	<0.001

R^2 ^= 0.11

Dependent variable: final mental component.

Variables excluded: size of the habitat, age, smoking, educational level, Body Mass Index.

## Discussion

This study shows that patients with stable COPD have, as compared to the reference population, a reduction of PCS-12, even in the initial stages, though they only show a reduction in MCS-12 in the most advanced phases of the disease. The determinant factors of both components of HRQoL include sex, FEV_1_, use of oxygen therapy and number of visits to emergency rooms and hospital admissions. In addition, the age, educational level and body mass index are also variables that influence the PCS-12.

The women in our study had lower HRQoL levels than men, both in the physical and mental component of quality of life. Several authors have reported that women usually suffer more respiratory symptoms than men [20], which could partly justify this finding. Furthermore, it has been demonstrated that, after adjusting for smoking, women show a higher risk of hospital admission for COPD than men [21].

Most previous studies have detected a mild to moderate association between the different areas of HRQoL and the degree of airflow obstruction [22-28]. In our study, a higher correlation has been found with the physical vs the mental component of HRQoL. Thus, it seems that the effects of COPD on health are not only mediated by the severity of the airflow limitation. For instance, it has been shown that the health condition perceived by the patients correlates better with the degree of dyspnea [29,30] or with the distance walked in the walking test [31] than with FEV_1_. The patients who obtain better results in these parameters report a limitation to fulfill their activities and the disease has less impact in their daily life.

The influence of hypoxemia and oxygen therapy on the HRQoL of patients with COPD has been assessed in previous studies. Some of them have found no relationship between HRQoL and the presence of respiratory failure [32,33]. On the contrary, we have observed that the use of oxygen therapy is a predictive factor independent from the health condition perceived by the patients. Similar results have been obtained in other studies. Thus, Okubadejo et al. [34] demonstrated, through a multivariate model, the existence of a relationship between HRQoL and the severity of hypoxemia. More recently, Ferreira et al. [35] have also demonstrated a marked worsening of HRQoL in patients with COPD receiving long-term oxygen therapy, with greater influence on physical and social function dimensions. Several reasons have been proposed to justify the trend of the patients to reject the treatment with oxygen therapy. These include the negative expectation linked to the use of an apparatus for long time periods to maintain health, the noise caused by the device, the mobility restriction resulting of its use, and the limitation involved by the fact that the patient must be confined at home most of the time [33,36,37].

Acute worsening is a common adverse event in the natural history of patients with COPD. It is the most common reason for medical visits, hospital admissions, and death in these patients [38]. Furthermore, there is an increasing evidence of the impact of these events on the health condition of patients with COPD. Thus, it has shown that patients suffering more than three annual acute worsening of COPD had a significantly greater impairment in their quality of life than those with a lower number of acute worsening [39,40]. Furthermore, Fan et al. [41] have shown that HRQoL is a predictive factor independent from hospitalization and mortality in these patients. In our study it has been also detected that the visits to emergency rooms and hospital admissions for acute worsening of the disease in the last year influence significantly the quality of life of patients with COPD, affecting both the physical and mental component.

The influence of age on HRQoL in patients with COPD is controversial. Some studies have detected no association between the two parameters [42] and others have demonstrated it through a logistic regression analysis that is an independent factor [23,43]. In our study we have only found a relationship with the physical component of quality of life, but not with the mental. The same has happened with other parameters such as the educational level of the patients evaluated. It is possible that subjects with a higher educational level have a greater purchasing power and have more material resources and, on the contrary, those with a lower level belong to the most disadvantaged classes, complaining later of health problems, have a higher environmental exposure, and the percentage of smokers among them is higher [43].

There is no agreement on the relationship between BMI and HRQoL. There seems to be an association between a poor nutritional status and quality of life worsening in patients with COPD, particularly those with emphysema [44]. Our results confirm that there is an association, though it is only demonstrated with the mental component of quality of life. The same has happened in another recent study, which only found a relationship with emotional function, though it was weak [43]. On the contrary, other authors have not evidenced any influence of bodyweight on the HRQoL of the patients with COPD [29,45].

Some limitations may have influenced the results of this study. First, we have used a generic questionnaire to measure HRQoL, less sensitive than the specific tools. However, the large sample size of our study enables to compensate this effect and give validity to our model. Second, all the patients were from primary care, so the results may not be extrapolable to all patients with COPD. However, a recent study evaluating this has found no relationship between HRQoL and the healthcare level [29].

In conclusion, the HRQoL of the patients with COPD is in part related to some parameters, including sex, airflow limitation, use of oxygen therapy and number of acute worsening and hospital admissions. However, these factors do not predict overall the high variability of HRQoL. Despite this, their measurement enables to identify the patients with a greater disability and enhances the assessment of the effects of various interventions on a standard basis.

## Competing interests

Javier Rejas Gutierrez and Antonio Martin Centeno are employees at Pfizer Spain and Elena Gobartt Vázquez is employee at Boehringer Ingelheim SA. The other authors have not any conflict of interest with Pfizer or Boehringer Ingelheim SA. This study has been funded by an unrestricted grant from Pfizer Spain and Boehringer Ingelheim SA.

## Authors' contributions

PCG, JRG, MGC, AGM and RJG have made substantive intellectual contributions to conception and design, acquisition of data and analysis and interpretation of data. PCG, JRG, JMD, AGM, and EGV have been envolved in drafting the manuscript and revising it critically for important intellectual content. All authors have given final approval of the version to be published.
